# Model selection for the extraction of movement primitives

**DOI:** 10.3389/fncom.2013.00185

**Published:** 2013-12-20

**Authors:** Dominik M. Endres, Enrico Chiovetto, Martin A. Giese

**Affiliations:** Section Computational Sensomotorics, Department of Cognitive Neurology, CIN, HIH, BCCN, University Clinic TübingenTübingen, Germany

**Keywords:** motor primitives, blind source separation, temporal smoothing, model selection, laplace approximation, bayesian methods, movement primitives

## Abstract

A wide range of blind source separation methods have been used in motor control research for the extraction of movement primitives from EMG and kinematic data. Popular examples are principal component analysis (PCA), independent component analysis (ICA), anechoic demixing, and the time-varying synergy model (d'Avella and Tresch, 2002). However, choosing the parameters of these models, or indeed choosing the type of model, is often done in a heuristic fashion, driven by result expectations as much as by the data. We propose an objective criterion which allows to select the model type, number of primitives and the temporal smoothness prior. Our approach is based on a Laplace approximation to the posterior distribution of the parameters of a given blind source separation model, re-formulated as a Bayesian generative model. We first validate our criterion on ground truth data, showing that it performs at least as good as traditional model selection criteria [Bayesian information criterion, BIC (Schwarz, 1978) and the Akaike Information Criterion (AIC) (Akaike, 1974)]. Then, we analyze human gait data, finding that an anechoic mixture model with a temporal smoothness constraint on the sources can best account for the data.

## 1. Introduction

In recent years substantial experimental evidence has been provided that supports the hypothesis that complex motor behavior is organized in modules or simple units called movement primitives (Flash and Hochner, 2005; Bizzi et al., 2008). In this framework each module, or motor primitive, consists of a set of movement variables, such as joint trajectories (Santello et al., 1998; Kaminski, 2007) or muscle activations (d'Avella et al., 2006; Chiovetto et al., 2010) acting synergistically over time. By combination of small numbers of these primitives complex motor behaviors can be generated. Several methods have been used so far in the literature for the identification of motor primitives starting from experimental data sets, which include both well-known classical unsupervised learning techniques based on instantaneous mixture models, such as principal component analysis (PCA) and independent component analysis (ICA) (Chiovetto et al., 2010; Dominici et al., 2011), or even more advanced techniques that include the estimation of temporal delays of the relevant mixture components (d'Avella et al., 2006; Omlor and Giese, 2011). On the one hand, all these approaches differ from each other in multiple aspects, such as their underlying generative models or the specific priors imposed on the parameters. On the other hand, however, for all of them the number of primitives to be extracted and subsequently used to approximate the original data has to be set a priori. To our knowledge only very few motor control studies have so far addressed the problem of model selection in a principled way, see e.g., Delis et al., (2013); Hart and Giszter, (2013) for notable exceptions. The existing generative models for the extraction of motor primitives have indeed been demonstrated to provide a low-dimensional decomposition of the experimental data, but no clear criterion has been developed to objectively determine which model is best suited for describing the statistical features of the data under investigation. We are concerned with two types of statistical features:

- “hard” constraints, such as the number of primitives. Determining this number is also known as “model order estimation.”
- “Soft” constraints, e.g., regularity measures. In other words, a constraint on a parameter is “soft,” if it expresses a preference or expectation for the parameter's value, but does allow for deviation from this preference given sufficient evidence. For example, when modeling human walking, we expect a periodic movement with predominantly low frequency components. However, higher frequency components might be critical to capture specific, more complex movement primitives. We therefore would like to allow for the possibility of overriding our initial expectations if the data indicate that this is appropriate. One such regularity measure, *temporal smoothness* quantified by a kernel function, is a novelty of our approach in the context of model selection for blind source separation in motor control.

Concerning the model order selection, several criteria have been developed. Most of them require the computation of the likelihood function (Schwarz, 1978; Akaike, 1987; Basilevsky, 1994; Minka, 2000; Zucchini, 2000) and attempt to determine the right model order as the one that offers the best trade-off between accuracy of data fitting and complexity of the model. Our approach uses this trade-off in a more general setting. Such information criteria were proven to identify with almost no error the model order of noisy data sets when these were corrupted with Gaussian noise, but performances were shown to be noticeably worse when data were corrupted with signal-dependent noise (Tresch et al., 2006), which is actually thought to affect strongly the neural control signals (Harris and Wolpert, 1998). In this article we present a new objective criterion for model-order selection that extends the other classical ones based on information-theoretic and statistical approaches. The criterion is based on a Laplace approximation of the posterior distribution of the parameters of a given blind source separation method, re-formulated as a Bayesian generative model. We derive this criterion for a range of blind source separation approaches, including for the first time the anechoic mixture model (AMM) described in Omlor and Giese (2011).

We provide a validation of our criterion based on an artificial ground truth data set generated in such a way to present well-known statistical properties of real kinematic data. We show in particular that our method performs at least as well as other traditional model order selection criteria [Akaike's Information Criterion, AIC (Akaike, 1974) and the Bayesian Information Criterion, BIC (Schwarz, 1978)], that it works for both instantaneous and delayed mixtures and allows to distinguish between these given moderately sized datasets, and that it can provide information regarding the level of temporal smoothness of the generating sources.

We finally apply the criterion to actual human locomotion data, to find that, differently from other standard synchronous linear models, a linear mixture of time shiftable components characterized by a specific degree of temporal smoothness is a better account of the data-generating process.

### 1.1. Related approaches

The well-known plug-in estimators, BIC and AIC, have the advantage of being easy to use when a likelihood function for a given model is available. Hence, they are often the first choice for model order estimation, but not necessarily the best one. In (Tu and Xu, 2011) several criteria for probabilistic PCA (or factor analysis) models were evaluated, including AIC, BIC, MIBS (Minka's Bayesian model selection) (Minka, 2000) and Bayesian Ying-Yang (Xu, 2007). The authors found that MIBS and Bayesian Ying-Yang work best. The approach presented in Kazianka and Pilz (2009) corrected the approximations made in MIBS, which yielded improved performance on small sample sizes. This corrected MIBS performed better than all other approaches tested in that paper, including AIC and BIC.

The authors of Li et al. (2007) estimated the number of independent components in fMRI data with AIC and minimum description length [MDL, (Rissanen, 1978)], which boils down to BIC. They showed that temporal correlations adversely affect the accuracy of standard complexity estimators, and proposed a sub-sampling procedure to remove these correlations. In contrast, we demonstrate below how to deal with temporal dependence as a part of our model. Another MDL-inspired approach, code length relative to a Gaussian prior (CLRG) was introduced in Plant et al. (2010) to compare different ICA approaches and model orders. It was demonstrated to work well on simulated data without the need of choosing additional parameters, such as thresholds, and it was shown that it is able to recover task-related fMRI components better than heuristic approaches.

Such heuristic approaches typically utilize some features of the reconstruction error (or conversely, of the variance-accounted-for (VAF)) as a function of the model order, e.g., finding a “knee” (inflection point) in that function, a procedure which is inspired by the scree test for factor analysis (Cattell, 1966). For example, the authors of Cheung and Xu (1999) experimented with an empirical criterion for ICA component selection. The independent components were ordered according to their contribution to the reduction of reconstruction error. Only those independent components were retained that had a large effect on this error. Similarly, the approach of Sawada et al. (2005) used “unrecovered power,” which is basically reconstruction error, to determine which components of a (reverberant) mixture are important. The work in Valle et al. (1999) compared various criteria for PCA component selection on real and simulated chemical reactor data, finding that some of the heuristic reconstruction-error based methods still perform well when PCA model assumptions are violated by the data-generating process.

To distinguish convolutive (but undelayed) mixtures from instantaneous ones, the work in Dyrholm et al. (2007) employed the framework of Bayesian model selection for the analysis of EEG data. Related to our approach, the authors of Penny and Roberts (2001) derived Laplace approximations to the marginal likelihood of several ICA model classes for model selection and model order determination. Their work is conceptually similar to our approach, but we also consider delayed mixtures.

All approaches reviewed so far are deterministic in nature. There are also sampling methods available for model selection purposes, see Bishop (2007) for details. One example is e.g., the work of Ichir and Mohammad-Djafari (2005) which used importance sampling and simulated annealing for model-order selection of L1-sparse mixtures.

## 2. Materials and methods

We develop our model (order) criterion in the framework of Bayesian generative model comparison Bishop (2007). Let *D* be observable data, Θ_*M*_ a tuple of model parameters for a model indexed by *M* (the “model index”) and Φ a tuple of hyperparameters. Using standard terminology, we denote

(1) $$\text{likelihood}:p\left(D|\Theta_{M},\Phi ,M\right)$$

(2) $$\text{prior}:p\left(\Theta_{M}|\Phi ,M\right).$$

The likelihood is the probability density of the data given the model parameters, model index and hyperparameters. The parameter prior is the probability density of the model parameters. Then the marginal likelihood of *M*, or model evidence for *M* is given by

(3) $$\begin{array}{l} p(D|\Phi ,M)=\int d\Theta_{M}p\left(D,\Theta_{M}|\Phi ,M\right)\\ \;=\int d\Theta_{M}p(D|\Theta_{M},\Phi ,M)p\left(\Theta_{M}|\Phi ,M\right) \end{array}$$

where the second equality follows from the product rule for probability distributions. Strictly speaking, the Φ would have to be integrated out as well after choosing a suitable prior for them. However, to keep the problem tractable we determine their value by maximizing the model evidence with respect to them, finding that this yields sufficiently good approximations for our purposes. Once we have evaluated Equation 3 for all *M*, we can select that *M* which maximizes the model evidence, since we have no *a-priori* preference for any *M*.

To apply this model selection framework, we reformulate three popular blind source separation (BSS) methods, namely probabilistic PCA (pPCA), ICA and anechoic demixing as generative models in section 2.1. This reformulation allows us to evaluate their likelihoods and parameter priors. We then use a Laplace approximation (Laplace, 1774) to compute an approximation to the marginal likelihood of each model. This approximation is derived in section 2.2.

### 2.1. Generative models of blind source separation methods

The BSS methods we consider all assume a linear generative model in discrete time, where observable data **X** can be written as a linear superposition of sources **S** multiplied by weights **W**. Let *t* = 1, …, *t* be the *T* (equally spaced) time points, *i* = 1, …, *I* the source index, and *j* = 1, …, *J* the signal index. Note that *J* could also be interpreted as a trial index, i.e., one signal repeated *J* times, or any combination of trials and signals. For the models we consider, there is no formal difference between “trial” and “signal,” as opposed to e.g., the time varying synergy model (d'Avella et al., 2006). Then **X** is a (*J* × *T*) matrix, **S** is (*I* × *T*) and consequently **W** must be (*J* × *I*) so that

(4) X=WS+Σ

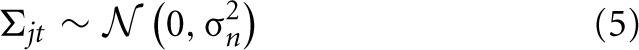
 where the entries of the noise matrix **Σ** are drawn independently from a Gaussian distribution with zero mean and variance σ^2^_*n*_. In an anechoic (delayed) mixture, the sources additionally depend on the signal index *j* (see section 2.1.3 for details).

The differences between the BSS approaches can be expressed as priors on **S** and **W**, which we describe in the following.

#### 2.1.1. Probabilistic PCA (pPCA)

PCA is one of the most widely used BSS approaches. In Tipping and Bishop (1999), it was demonstrated how PCA results from a probabilistic generative model: assuming the data have mean zero (i.e., ∀*j* : ∑_*t*_ **X**_*jt*_ = 0), and using an independent zero-mean Gaussian prior on the sources, i.e.,

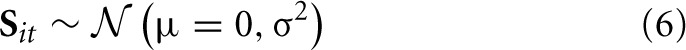



the weights **W** which maximize the marginal likelihood of **X** after integrating out **S**, are given by the scaled (and possibly rotated) principal *I* eigenvectors of the (*J* × *J*) data covariance matrix, $\frac{1}{T}XX^{T}$. This model differs from PCA insofar as the sources will only be equal to the PCA factors in the noise-free limit σ_*n*_ → 0, and is hence referred to as *probabilistic* PCA (Tipping and Bishop, 1999). Similarly, when we put a prior on the weights

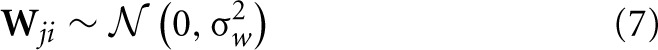



and integrate them out, we find that the best *I* sources **S** are the principal eigenvectors of the (*T* × *T*) data covariance matrix $\frac{1}{J}X^{T}X$ (assuming zero mean signals at every time step, i.e., ∀*t* : ∑_*j*_ **X**_*jt*_ = 0). We will therefore use both priors for a completely probabilistic pPCA model^1^.

**Figure 1:**
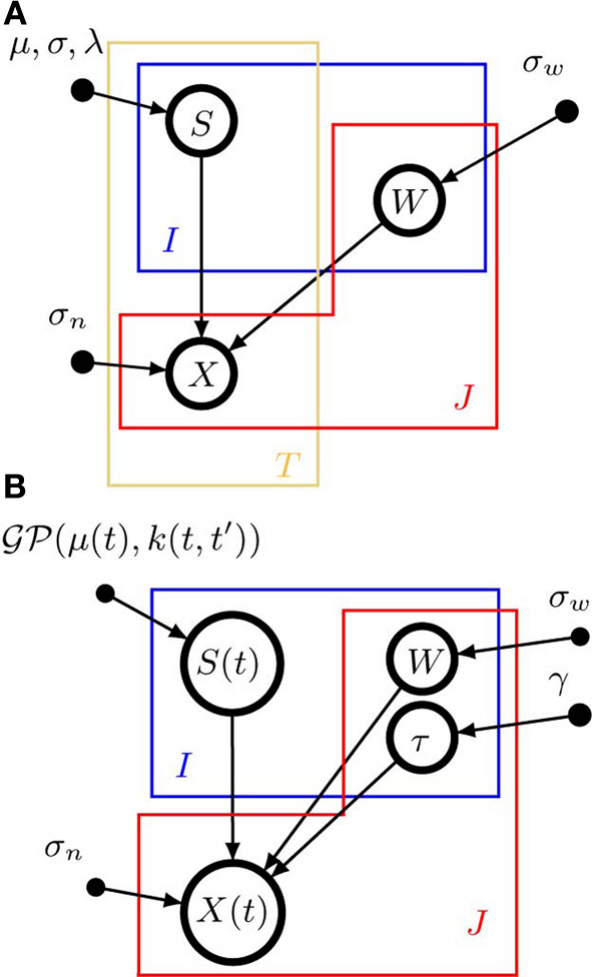
**Graphical model representations of the blind source separation algorithms for which we compute a model evidence approximation**. We follow standard graphical modeling terminology (see e.g., Bishop, 2007). Open circles represent random variables, which may also be random functions. Filled circles are parameters. Arrows denote conditional dependencies. The *plates* (colored frames) indicate that the enclosed structure is repeated as often as the corresponding letter indicates. Enclosure in multiple plates indicates a product of repetitions. For example, in panel **(A)** there are *I* × *T* random variables *S* which comprise the source matrix. **(A)** Instantaneous, undelayed mixtures such as pPCA (where λ = 0) and ICA. *J* × *T* signals *X* are computed by mixing *I* × *T* sources *S* with *J* × *I* weights *W*. σ_*w*_ is the standard deviation of the zero-mean Gaussian prior on the weights. σ_*n*_ is the noise standard deviation. μ and σ are the parameters of the Gaussian part of the prior on the sources, λ measures the deviation from Gaussianity. **(B)**. Convolutive, delayed mixtures, like the anechoic mixture of Omlor and Giese (2011) with additional temporal smoothness constraints. *I* source functions *S*(*t*) are drawn from a Gaussian process 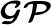
(μ(*t*), *k*(*t*, *t*′)) with mean function μ(*t*) and kernel *k*(*t*, *t*′). These sources are shifted in time by *J* × *I* many delays (one per trial and source) drawn from an exponential distribution with parameter γ and mixed with *J* × *I* weights *W* which are drawn from a zero-mean Gaussian distribution with standard deviation σ_*w*_, to yield *J* signals *X*(*t*). For details, see text.

A graphical model representation of pPCA is shown in Figure 1A. Open circles represent random variables, which may also be random functions. Filled circles are parameters. Arrows denote conditional dependencies. The *plates* (colored frames) indicate that the enclosed structure is repeated as often as the corresponding letter indicates. Enclosure in multiple plates indicates a product of repetitions. Thus, in a pPCA model, *I* × *T* sources are *a-priori* drawn independently of each other (μ and σ are parameters, not random variables), and source values have no dependencies across time. Likewise, weights have no dependencies across sources or signals. In contrast, data points depend on both weights and sources, as indicated by the arrows converging on **X** from **S** and **W**.

Given the generative model (Equation 4) and the prior specification (Equation 6 and Equation 7), we can now write down the likelihood and prior terms which we need for the evaluation of the model evidence (Equation 3). To this end, we identify the number of sources *I* with the model index *M*, and (cf. Equation 3)

(8) D=X

(9) $$\Theta_{M}=(W,S)$$

(10) $$\Phi =\left(\mu ,\sigma ,\sigma_{w},\sigma_{n}\right)$$

(11) $$p\left(D|\Theta_{M},\Phi ,M\right)=\frac{\exp\left(-\frac{1}{2\sigma_{n}^{2}}{\left\|X-WS\right\|}_{F}\right)}{{\sqrt{2\pi \sigma_{n}^{2}}}^{JT}}$$

(12) $$p(\Theta_{M}|\Phi ,M)=\frac{\exp\left(-\frac{1}{2\sigma^{2}}\|S_{it}-\mu \cdot 1_{IT}{\|}_{F}\right)}{{\sqrt{2\pi \sigma^{2}}}^{IT}}\times \frac{\exp\left(-\frac{1}{2\sigma_{w}^{2}}\|W{\|}_{F}\right)}{{\sqrt{2\pi \sigma_{w}^{2}}}^{JI}}$$

where **1**_*IT*_ is an (*I* × *T*) matrix with every element being 1, and ‖**A**‖_*F*_ is the Frobenius norm of matrix **A**.

#### 2.1.2. Independent component analysis (ICA)

The term ICA refers to a variety of BSS methods which try decompose signals into sources with two main goals:

1. The sources are as statistically independent as possible according to some suitably chosen measure, and
2. the sources allow for a good reconstruction of the signals.

Infomax ICA (Bell and Sejnowski, 1995) tries to achieve these goals by maximizing the mutual information (Cover and Thomas, 1991) between sources and signals, which clearly promotes the second goal. The first one is promoted if the BSS system contains an information bottleneck, e.g., fewer sources than signals. In that case, maximizing mutual information amounts to maximizing the total source entropy, which is achieved if the sources are independent.

The FastICA algorithm (Hyvarinen, 1999) aims directly at minimizing the mutual information between the sources, thereby promoting goal one. Goal 2 is achieved by constraining the (linear) transformation from signals to the sources to be invertible, or at least almost invertible in the noisy or lossy case, such that the signals can be reconstructed using the generative model above (Equation 4). Mutual information is measured via *negentropy*, which is the negative difference between the entropy of a source and the entropy of a variance-matched Gaussian variable, i.e., it is a measure of non-Gaussianity. Maximizing negentropy then minimizes mutual information. To measure negentropy, the authors of Hyvarinen (1999) used the “contrast function” approach developed in Hyvärinen (1998). Contrast functions provide constraints on expectations of probability distributions, in addition to the mean and variance constraints of Gaussians. Consequently, the maximum entropy distributions obeying these constraints have the contrast function(s) as sufficient statistics, with an associated natural parameter, which controls the deviation of the resulting distribution from a Gaussian. For a detailed derivation see Hyvärinen (1998). This motivates the following source prior for probabilistic ICA models: let *G*(.) be the contrast function, then

(13) $$p\left(S_{it}\right)=\frac{1}{Z(\mu ,\sigma ,\lambda )}\exp\left(-\frac{1}{2\sigma^{2}}{\left(S_{it}-\mu \right)}^{2}+\lambda G(S_{it}-\mu )\right)\;\;$$

where λ is the natural parameter associated with *G*(.). The normalization constant *Z*(μ, σ, λ) can be evaluated by numerical integration, since the prior is a density over a one-dimensional random variable. Similar to pPCA, we use a Gaussian prior on the weights. The graphical model representation of ICA is the same as for pPCA (see Figure 1A), since there is no a-priori dependency between sources or weights across time.

We can now identify the number of sources *I* with the model index *M*, and furthermore (cf. Equation 3)

(14) D=X

(15) $$\Theta_{M}=(W,S)$$

(16) $$\Phi =\left(\mu ,\sigma ,\sigma_{w},\sigma_{n},\lambda \right)$$

(17) $$p(D|\Theta_{M},\Phi ,M)=\frac{\exp\left(-\frac{1}{2\sigma_{n}^{2}}{\left\|X-WS\right\|}_{F}\right)}{{\sqrt{2\pi \sigma_{n}^{2}}}^{JT}}$$

(18) $$p(\Theta_{M}|\Phi ,M)=\prod_{i,t}p(S_{it})\times \frac{\exp\left(-\frac{1}{2\sigma_{w}^{2}}\|W{\|}_{F}\right)}{{\sqrt{2\pi \sigma_{w}^{2}}}^{JI}}$$

#### 2.1.3. Anechoic mixture models (AMM) and smooth instantaneous mixtures (SIM)

AMMs may be seen as an extension of the above BSS approaches to deal with time-shifted sources (Omlor and Giese, 2007a). Such time shifts are obviously useful in motor control, where coordinated movement patterns, such as gaits, might be characterized by opposite joints moving in a similar manner but time-shifted against each other (e.g., the legs during walking); the well-known time-varying synergy model (d'Avella et al., 2006) is a kind of AMM. The generative models of AMMs are linear with additive Gaussian noise (similar to Equation 4), but the sources *S*_*i*_(*t*) are shifted by delays τ_*ji*_, which are the elements of a (*J* × *I*) matrix τ. We draw these delays from an exponential prior with mean γ, which promotes delays that differ sparsely from zero.

(19) $$\begin{array}{l} X_{jt}=\sum_{i}W_{ji}S_{i}(t-\tau_{ji})+\eta_{jt}\\ \;=\sum_{i}\hat{X}+\eta_{jt} \end{array}$$

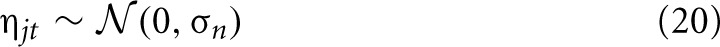

(21) $$p(\tau_{ji})=\gamma \exp\left(-\frac{\tau_{ij}}{\gamma }\right)$$

where we define the matrix of the reconstructed signals $\hat{X}$ as ${\hat{X}}_{ji}=\sum_{i}W_{ji}S_{i}(t-\tau_{ji})$. Moreover, we impose soft temporal regularity constraints on the sources. To this end, we draw the sources from a Gaussian process (GP) (Rasmussen and Williams, 2006) with mean function μ(*t*) and covariance (or kernel) function *k*(*t*, *t*′). A GP is a prior over functions *S*(*t*) where the joint distribution of any finite number of function values at times *t*_1_, …, *t*_*N*_ follows a multivariate Gaussian distribution i.e.,

(22) $$\vec{S}=(S(t_{1}),\ldots ,S(t_{N}))$$

(23) $$\vec{\mu }=\left(\mu (t_{1}),\ldots ,\mu (t_{N})\right)$$

(24) $$K_{mn}=k(t_{m},t_{n})$$

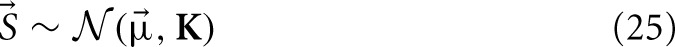


**Figure 2:**
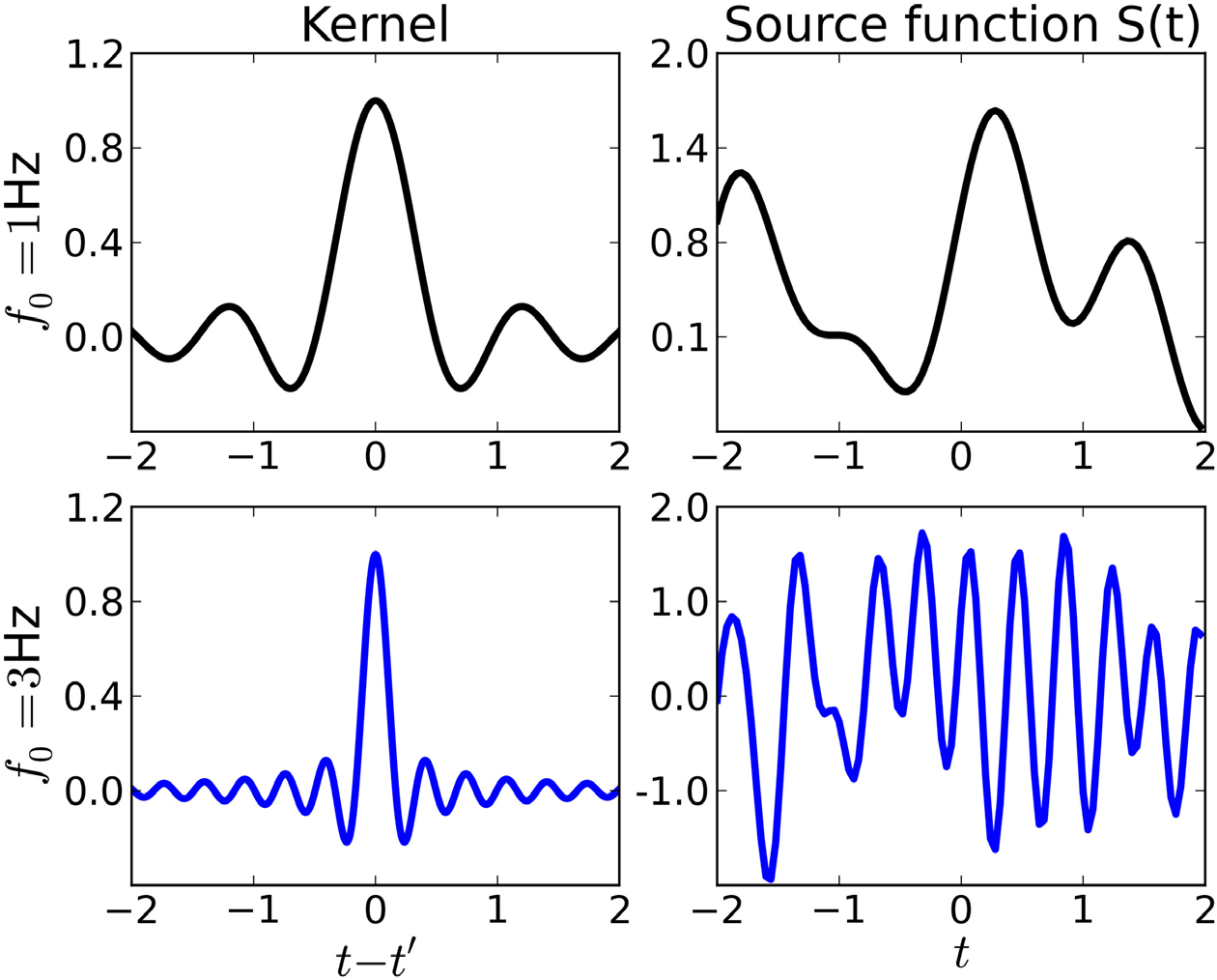
**Examples of kernel functions (left) and sources (right) drawn from a Gaussian process prior with the corresponding kernel**. Throughout this paper, we use shift-invariant kernels of the form *k*(*t*, *t*′) ∝ sinc(2*f*_0_|*t* − *t*′|). **Top row**: Kernel function for *f*_0_ = 1Hz (left) and source function drawn from a Gaussian process with that kernel. The source varies rather smoothly on a timescale comparable to *f*_0_. **Bottom row**: Kernel function and source for *f*_0_ = 3Hz.

Thus, the choice of kernel function determines how much the function values at different points tend to co-vary a priori. Throughout this paper, we will use kernel functions of the form

(26) $$k(t,t^{\prime })\propto \text{sinc}(2f_{0}|t-t^{\prime }|)=\frac{\sin(2\pi f_{0}|t-t^{\prime }|)}{2\pi f_{0}|t-t^{\prime }|}$$

which is also called *wave kernel* (Genton, 2001) in the machine learning literature. This choice is motivated by the observation that the inverse Fourier transform of an ideal low-pass filter with cutoff-frequency *f*_0_ is proportional to this kernel. Thus, functions drawn from a GP with this kernel will vary on timescales comparable to *f*_0_, see Figure 2 for examples. Note, however, that the regularization provided by the kernel is “soft”: when learning sources from small datasets, they will have the smoothness properties given by the kernel. For large datasets, the kernel regularization may be overridden by the data.

With this prior, the matrix of reconstructed signals $\hat{X}$ and using as model index the tuples *M* = (*I*, *f*_0_) we find

(27) D=X

(28) $$\Theta_{M}=(W,\tau ,S_{1}(t),\ldots ,S_{I}(t))$$

(29) $$\Phi =\left(\mu (t),k_{f_{0}}(t,t^{\prime }),\sigma_{n},\sigma_{w}\right)$$

(30) $$p(D|\Theta_{M},\Phi ,M)=\frac{\exp\left(-\frac{1}{2\sigma_{n}^{2}}\|X-\hat{X}{\|}_{F}\right)}{{\sqrt{2\pi \sigma_{n}^{2}}}^{JT}}$$

(31) $$\begin{array}{l} p(\Theta_{M}|\Phi ,M)=\frac{\exp(-\frac{1}{2}S_{i}K^{-1}S_{i}^{T})}{{\sqrt{2\pi }}^{T}\sqrt{\left|K\right|}}\prod_{j,i}\gamma \exp\left(-\frac{\tau_{ji}}{\gamma }\right)\\ \;\times \frac{\exp\left(-\frac{1}{2\sigma_{w}^{2}}\|W{\|}_{F}\right)}{{\sqrt{2\pi \sigma_{w}^{2}}}^{JI}} \end{array}$$

where **S**_*i*_ is the i-th row of the *undelayed* source matrix, i.e., the matrix of the source functions sampled at times *t* = 1, …, *T*. A graphical model representation of AMM is shown in Figure 1B.

As a special case of the AMM model above, we consider the case ∀*i*, *j* : τ_*ji*_ = 0, i.e., a mixture without delays, but GP-induced temporal regularization. In the following, we refer to this as the *smooth instantaneous mixture*, or SIM.

### 2.2. Laplace approximation

We now turn to the evaluation of the model evidence, Equation 3. The difficult part, as usual in Bayesian approaches, is the integral over the model parameters Θ (we drop the index *M* in the following for notational simplicity, since we evaluate the model evidence for each *M* separately). Instead of an exact solution, we therefore resort to a *Laplace approximation* (Laplace, 1774; Bishop, 2007). To use this approach, concatenate the Θ into a vector and then construct a saddle-point approximation (Reif, 1995) of intractable integrals of the form

(32) ∫dΘexp(−f(Θ))

assuming that *f*(Θ) has a single, sharply peaked minimum at some Θ^*^ = argmin_Θ_*f*(Θ) and is twice continuously differentiable. In this case, only exponents close to the minimal exponent *f*(Θ^*^) will make noticeable contributions to the integral. Hence, we can approximate *f*(Θ) *locally* around Θ^*^ by a Taylor expansion

(33) $$f(\Theta )\approx f(\Theta^{*})+\nabla_{\Theta^{*}}f{\left(\Theta \right)}^{T}(\Theta -\Theta^{*})+\frac{1}{2}{\left(\Theta -\Theta^{*}\right)}^{T}H\;(\Theta -\Theta^{*})$$

where

$$H_{uv}={\frac{\partial^{2}f(\Theta )}{\partial \theta_{u}\partial \theta_{v}}|}_{\Theta^{*}}$$

is the Hessian matrix of the 2nd derivatives evaluated at Θ^*^. Since Θ^*^ is the location of the minimum of *f*(Θ), it follows that ∇_Θ^*^_f(Θ)^*T*^ = 0 and **H** is positive (semi-)definite. Thus

(34) $$f(\Theta )\approx f(\Theta^{*})+\frac{1}{2}{\left(\Theta -\Theta^{*}\right)}^{T}H\;(\Theta -\Theta^{*})$$

and we can approximate the integral as

$$\begin{array}{l} \;\int d\Theta \exp\left(-f(\Theta )\right)\\ \approx \int d\Theta \exp\left(-f(\Theta^{*})-\frac{1}{2}{\left(\Theta -\Theta^{*}\right)}^{T}H\;(\Theta -\Theta^{*})\right)\\ =\exp\left(-f(\Theta^{*})\right)\int d\Theta \exp\left(-\frac{1}{2}{\left(\Theta -\Theta^{*}\right)}^{T}H\;(\Theta -\Theta^{*})\right)\\ =\exp\left(-f(\Theta^{*})\right)\frac{{\left(2\pi \right)}^{\frac{F}{2}}}{\sqrt{\left|H\right|}} \end{array}$$

where *F* = dim(Θ) is the dimensionality of Θ. For the derivation of our model comparison criterion, we will need the logarithm of this integral:

(35) $$\log\left(\int d\Theta \exp\left(-f(\Theta )\right)\right)\approx -f(\Theta^{*})+\frac{F}{2}\log(2\pi )-\frac{1}{2}\log\left(\left|H\right|\right).$$

In summary, the Laplace approximation replaces the intractable integral with differentiation, which is always possible for the models we consider.

To approximate the model evidence (Equation 3) in this way, let

(36) $$\Theta^{*}={\text{argmin}}_{\Theta }\left[-\log(p(D|\Theta ,\Phi ,M))-\log(p(\Theta |\Phi ,M))\right]$$

in other words, Θ^*^ are the parameters which maximize the likelihood subject to the regularization provided by the parameter prior. Furthermore, denote

(37) $$\begin{array}{l} H_{uv}=-{\frac{\partial^{2}\log(p(D|\Theta ,\Phi ,M))}{\partial \Theta_{u}\Theta_{v}}|}_{\Theta^{*}}\\ \;-{\frac{\partial^{2}\log(p(\Theta |\Phi ,M))}{\partial \Theta_{u}\Theta_{v}}|}_{\Theta^{*}} \end{array}$$

and thus

(38) $$\begin{array}{l} p(D|\Phi ,M)\approx \underset{\text{log-likelihood}}{\underset{︸}{\log(p(D|\Theta^{*},\Phi ,M))}}+\underset{\text{log-prior}}{\underset{︸}{\log(p(\Theta^{*}|\Phi ,M))}}\\ \;+\underset{\text{log-posterior-volume}}{\underset{︸}{\frac{\text{dim}(\Theta )}{2}\log(2\pi )-\frac{1}{2}\log(|H|)}} \end{array}$$

which we will refer to as the *LAP* criterion for model comparison: the larger LAP, the better the model. It comprises three parts, which can be interpreted: the log-likelihood measures the goodness of fit, similar to explained variance or VAF. The second term is the logarithm of the prior, which corresponds to a regularization term for dealing with under-constrained solutions for Θ when the datset is small. Finally, the third part measures the volume of the parameter posterior, since **H** is the posterior precision matrix (inverse covariance) of the parameters in the vicinity of Θ^*^, i.e., it indicates how well the data constrain the parameters (large |**H**| means small posterior volume, which means Θ is well-constrained).

We will compare the LAP criterion to two standard model complexity estimators below (see section 3): BIC and AIC. BIC is given by

(39) $$\text{BIC}=-2\left(\log(p(D|\Theta^{*},\Phi ,M))-\frac{1}{2}\text{dim}(\Theta )\log(N)\right)$$

where *N* is the number of data-points. The best model is found by minimizing BIC w.r.t. *M*. BIC can be obtained from LAP in the limit *N* → ∞, by dropping all terms from LAP which do not grow with *N* and multiplying by −2. Assuming that the model has no latent variables (whose number typically grows with *N*), the terms to be dropped from Equation 38 are the log-prior, the first term of the posterior volume, and the second term of the Hessian (Equation 37). For i.i.d. observations, the determinant of the first term of the Hesssian will typically grow like *Nc*^dim(Θ)^ where *c* is some constant independent of *N*. Hence, the BIC follows. While this reasoning is somewhat approximate (a rigorous derivation can be found in Schwarz (1978)), it highlights that we might expect LAP to become more similar to BIC as the dataset increases.

AIC is originally derived from information-theoretic arguments (Akaike, 1987): a good model loses only a small amount of information when approximating (unknown) reality. When information is measured by Kullback-Leibler divergence (Cover and Thomas, 1991), AIC follows. Alternatively, it also obtained by choosing a model complexity prior which depends on *N* and dim(Θ) (Burnham and Anderson, 2004) and is given by

(40) $$\text{AIC}=-2\left(\log(p(D|\Theta^{*},\Phi ,M))-\text{dim}(\Theta )\right).$$

Like BIC, a good model has a low AIC score.

### 2.3. Assessment of criterion performance

To validate our criterion we assessed its performance on synthesized data sets with well-known statistical properties and on actual kinematic data collected from human participants during a free walking task. We also compared the results with those provided by AIC and BIC. Before applying the model selection criteria we factorized each available data set according to the mixture models Equation 4 and Equation 19. The identification of the parameters Θ was carried out in two phases: first, we applied singular value decomposition to identify the principal components (for PCA), or fastICA (Hyvarinen, 1999) or the anechoic demixing algorithm mentioned above (Omlor and Giese, 2011) to yield weights and sources. Second, we used these solutions to initialize an optimization of the corresponding likelihood function, to determine the optimal parameters Θ^*^ and hyperparameters Φ needed for the Laplace approximation. The optimization in the second step was carried out using the L-BFGS-B routine in the SciPy package (Jones et al., 2001) for Θ^*^, Φ was then re-estimated for fixed Θ^*^. This second optimization was necessary for two reasons: the statistical reformulations of pPCA and ICA will yield solutions which are very similar, but not identical to the original algorithms, and the AMM method from Omlor and Giese (2011) can not handle temporal smoothness priors. The number of components *I* identified ranged, for all algorithms, from 1 to 8.

#### 2.3.1. Ground-truth data generation

We simulated kinematic-like data (mimicking, for instance, joint-angle trajectories) based on the generative models Equation 4 and Equation 19 that is linear combinations of *I* primitives that could be synchronous (SIM) or shifted in time (AMM). For the generation of each primitive *S*_*i*_(*t*) we drew 100 random samples from a normal distribution (MATLAB (2010) function “randn”) and then we low-pass filtered them with a 6th-order Butterworth filter [MATLAB (2010) functions “butter” and “filtfilt”]. Two cut-off frequencies were used for filtering, respectively, 5 and 10 Hz, to simulate data with two different frequency spectra. Sampling frequency of the data was assumed to be 100 Hz. This procedure allowed to generate band-limited sources mimicking actual kinematic or kinetic trajectories of time duration *T* = 1 s. We generated artificial mixture data by combining a number of sources ranging from 1 to 4. Combination coefficients of the mixing matrix **W** were generated from a uniform continuous distribution in the interval [−10,10]. Temporal delays τ_*ji*_ were drawn, when needed, from an exponential distribution of mean 20. Sets of noisy data were generated by corrupting noiseless data generated as described above with signal dependent noise. Noise was drawn from a Gaussian distribution of variance σ = α|*x*_*i*_(*t*)|, where α is the slope of the relationship between the standard deviation and the noiseless data values *x*_*i*_(*t*) (Sutton and Sykes, 1967; Schmidt et al., 1979; van Beers et al., 2004). The slope α was computed though an iterative procedure. Starting from α = 0, its value was iteratively increased by a predefined increment until a desired noise-level 1 − *R*^2^ was reached and stayed constant for at least 10 consecutive computations of 1 − *R*^2^ given the same value of α. We define *R*^2^ as follows: as the artificial noiseless data sets and their corresponding noisy versions are multivariate time-series, a measure of similarity (typically a ratio of two variances) must be defined using a multivariate measure of data variability. We used the “total variation” (Mardia et al., 1979), defined as the trace of the covariance of the signals, to define a multivariate measure as follows:

(41) $$R^{2}=1-\frac{{\left\|X_{noiseless}-X_{noisy}\right\|}^{2}}{{\left\|X_{noiseless}-{\bar{X}}_{noiseless}\right\|}^{2}}$$

where **X**_noiseless_ is the matrix of the noiseless data set, **X**_noisy_ the noisy data, and where **X**_noiseless_ is a matrix with the mean values of the noiseless data over trials. For each noiseless data set, two datasets were generated with 1 − *R*^2^ levels equal to 0.15 and 0.3, corresponding to approximate signal-to-noise ratios of 22 dB and 15 dB, respectively. We thus generated 2 models (AMM/SIM) x 2 cut-off frequencies (5 Hz/10 Hz) × 4 number of sources x 3 levels of noise = 48 different data sets. Each of those datasets contained *J* ∈ {5; 10; 25} (data) trials. A “trial” (one row of the matrix **X** in Equation 4) is a one-dimensional time-series sampled at *T* points in time. For reliable averages, we drew 20 data sets for each number of trials.

#### 2.3.2. Actual kinematic data

We applied the model selection criteria to select also the model of a second data set consisting of movement trajectories of human actors walking neutrally, or with different emotional styles (happy and sad). This data was originally recorded for the study presented in Roether et al. (2008). The movements were recorded using a Vicon (Oxford, UK) optoelectronic movement recording system with 10 infrared cameras, which recorded the three-dimensional positions of spherical reflective markers (2.5 cm diameter) with spatial error below 1.5 mm. The 41 markers were attached with double-sided adhesive tape to tight clothing, worn by the participants. Marker placement was defined by the Vicon's PlugInGait marker set. Commercial Vicon software was used to reconstruct and label the markers, and to interpolate short missing parts of the trajectories. Sampling rate was set at 120 Hz. We recorded trajectories from six actors, repeating each walking style three times per actor. A hierarchical kinematic body model (skeleton) with 17 joints was fitted to the marker positions, and joint angles were computed. Rotations between adjacent body segments were described as Euler angles, defining flexion, abduction and rotation about the connecting joints. The data for the BSS methods included only the flexion angles of the lower body joints, specifically right and left pelvis, hips, knees and ankles, since the other angles had relatively high noise levels. From each trajectory only one gait cycle was extracted, which was time normalized. This resulted in a data set with 432 samples with a length of 100 time points each. It was already shown previously (Omlor and Giese, 2007a,b) that an anechoic mixture model is more efficient than synchronous models for the representation of such kinematic data. To test the capability of the new LAP criterion to confirm such an observation we applied temporal shift to each trajectory of the data set. Each delay corresponding to a specific trajectory was drawn from a continuous uniform statistical distribution in the interval [−20,20], the sign of the delay determining the shift direction (forwards or backwards), signals were wrapped around at the boundaries of the 100 time-point interval.

## 3. Results

We first present the evaluation of the three model selection criteria, LAP, BIC and AIC on the ground truth data described above in section 2.3.1. The evaluation is done with respect to three questions: how well can the generator type be detected (AMM or SIM), how accurate is the number of sources *I* estimation, and whether the amount of temporal smoothness [i.e., *f*_0_ in Equation 26] can be determined. Second, we analyze the human gait data.

### 3.1. Ground truth evaluation

#### 3.1.1. Model type detection

We measure the accuracy with which the generating model can be detected by the classification rate, averaged across generating and estimated number of sources, the estimated *f*_0_ and the 20 data sets per condition. It is given by

(42) $$\text{classification rate}=\frac{\text{number of correct detections}}{\text{total number of trials}}$$

**Figure 3:**
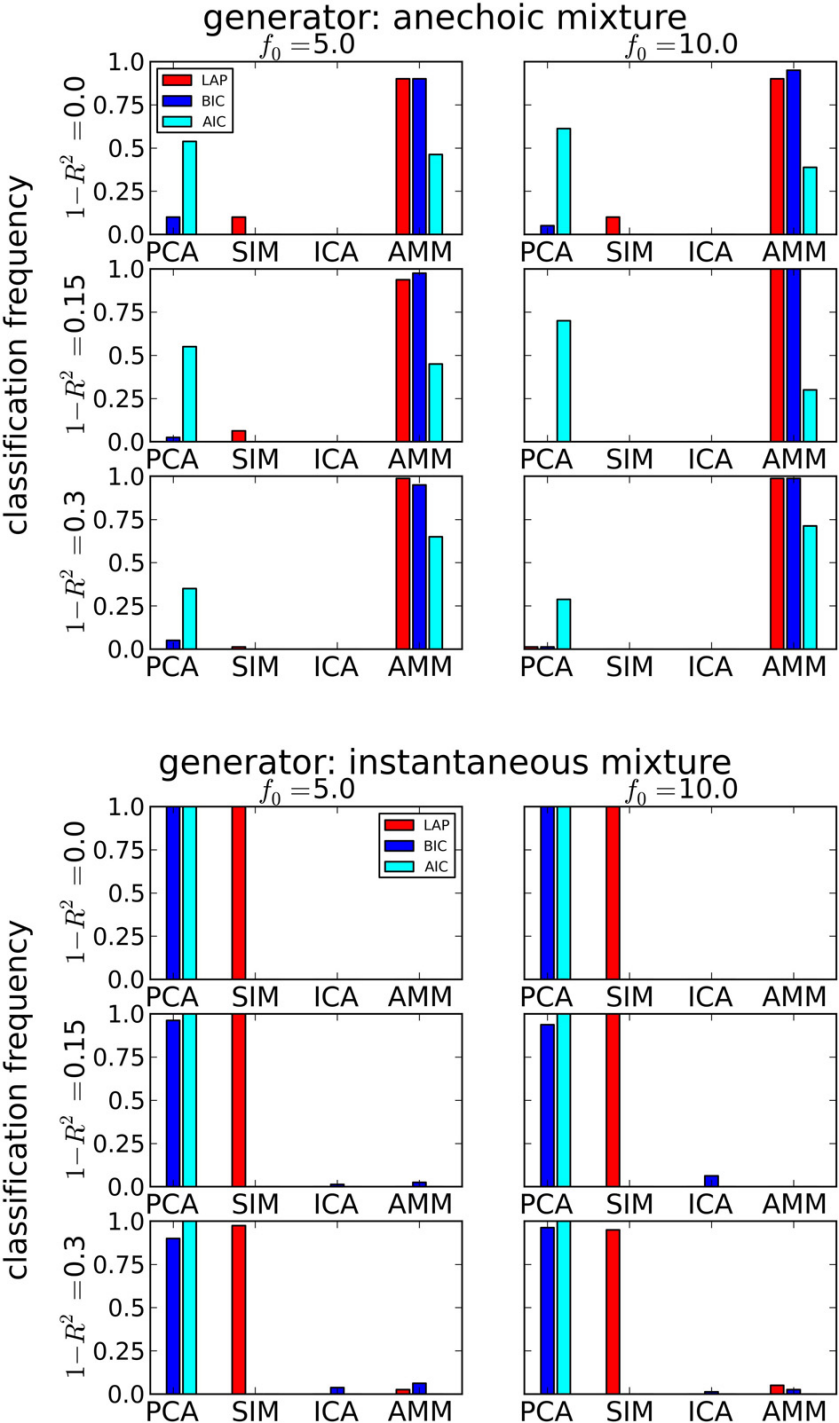
**Determining if the model type is a smooth instantaneous mixture (SIM) or an anechoic delayed mixture (AMM) from 10 trials, after marginalizing the estimated number of sources**. **Top**: Anechoic ground truth. BIC and LAP perform comparably well, AIC often mistakes an AMM for a pPCA model. **Bottom**: Ground truth from smooth instantaneous mixture. Only the Laplace approximation criterion (LAP) correctly detects the SIM model in the majority of cases, BIC and AIC confuse SIM with pPCA due to their inability of handling soft constraints. For details, see text.

The results are summarized in Table 1 for each number of trials *J*. LAP clearly outperforms BIC and AIC, particularly for small *J*. To understand where this difference comes from, Figure 3 shows a detailed analysis of the results for *J* = 10 trials. The anechoic generator is correctly detected by both LAP and BIC in most cases, whereas AIC often mistakes it for a pPCA model. The SIM generator, on the other hand, is only detected by LAP, both BIC and AIC mistake it for a pPCA model. Hence, LAP achieves very high classification rates, BIC is wrong about half the time, and AIC is even worse. This is due to the terms in BIC and AIC which punish complex models (second terms in Equation 39 and Equation 40, respectively): they only depend on the *number* of degrees of freedom and the number of data-points, but do not measure the effects of any “soft” constraints. Since such soft constraints will have a reducing effect on the likelihood, BIC and AIC will prefer models without such soft constraints over those with constraints. Consequently, BIC and AIC select pPCA over SIM. Note that in the limit of *f*_0_ → ∞, the kernel of the SIM model will give rise to a diagonal covariance matrix **K**, and thus uncorrelated sources, whereas the **K** for finite *f*_0_ will impose a correlational constraint. Thus, the SIM model will turn into a pPCA model in this limit.

**Table 1:**
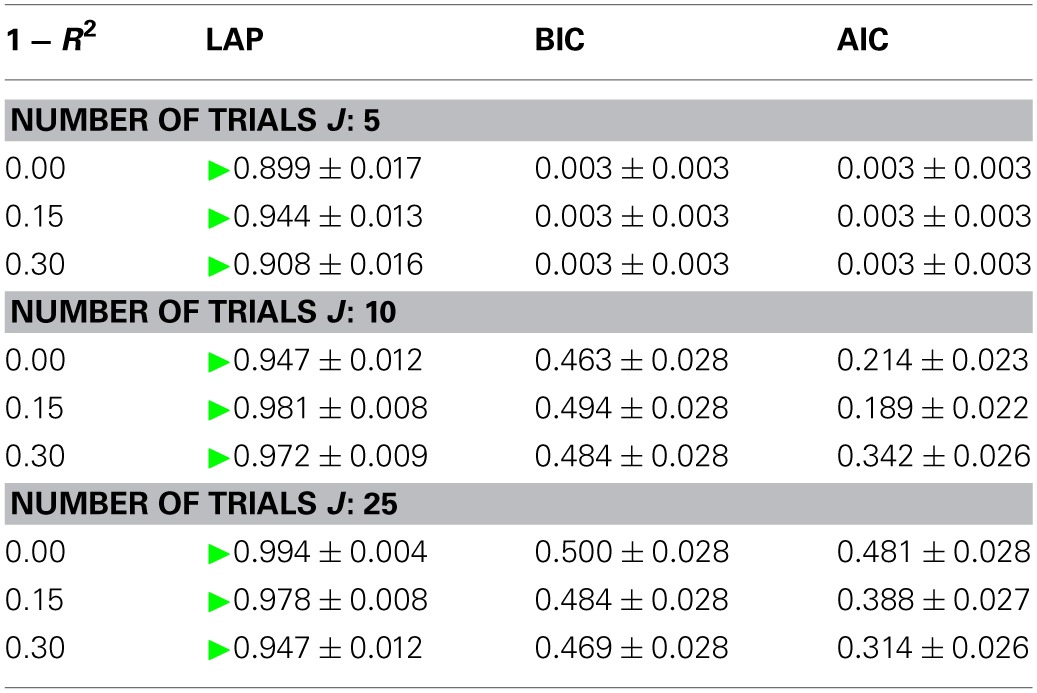
**Model type classification rates of the three tested criteria, for number of trials between 5 (top) and 25 (bottom)**.

*1 − R^2^ is the noise level from Equation 41*.

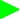
 *indicates the best criterion for each row. LAP consistently outperforms BIC and AIC, mostly because the latter two are unable to distinguish between a smooth instantaneous mixture and a pPCA model (see also Figure 3). Furthermore, for 5 trials BIC and AIC tend mistake an anechoic mixture for an ICA model, leading to model type classification rates which are virtually zero*.

In contrast, the LAP criterion measures the effect of the source correlations via the log-prior and log-posterior-volume terms. If the posterior is concentrated in a region of parameter space where the prior is high, the effects of the reduced likelihood can be counterbalanced. Since we evaluated the LAP criterion for *f*_0_ ∈ {5 Hz,10 Hz}, one of the tested SIM models will match the generator and have a correspondingly high LAP score.

#### 3.1.2. Estimating the number of sources

Next, we looked at how well the criteria are suited for estimating the number of sources. Table 2 shows the average difference between estimated and generating number of sources, averaged across noise levels, number of generating sources and *f*_0_s. An empty cell indicates that this model would have been picked by the above model type detection only very infrequently.

**Table 2:**
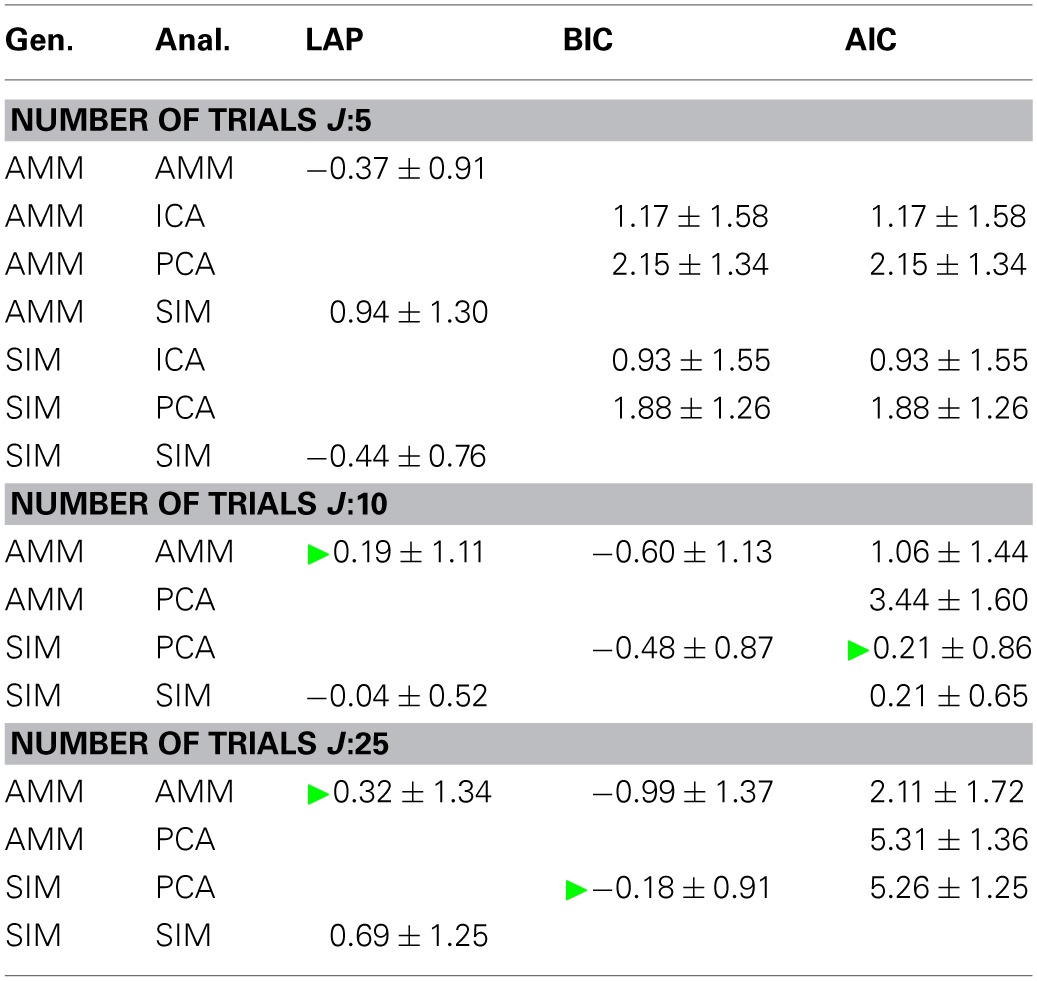
**Estimating the number of sources, marginalized across all noise levels and cutoff frequencies *f*_0_**.

*Shown is the difference between the best number of sources determined with a given criterion (LAP, BIC, or AIC) and the number of sources in the generator. Gen. is the generating model, either anechoic (AMM) or smooth instantaneous (SIM) mixture. Anal. is the analysis algorithm. An empty cell indicates that a model comparison criterion (LAP, BIC, AIC) would have picked the corresponding analysis algorithm with a chance of less than 10% (cf. Figure 3 and Table 1). For rows with more than one entry*, 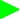 *indicates best criterion*.

Particularly for a small number of trials *J*, LAP is closer to the correct number of sources than BIC or AIC. For larger number of trials, the results between BIC and LAP become more similar, which is to be expected, even though BIC does not detect the correct model type. Moreover, the average number of sources estimated by LAP is always within one standard deviation of 0, and these standard deviations are mostly smaller than those of BIC and AIC.

**Figure 4:**
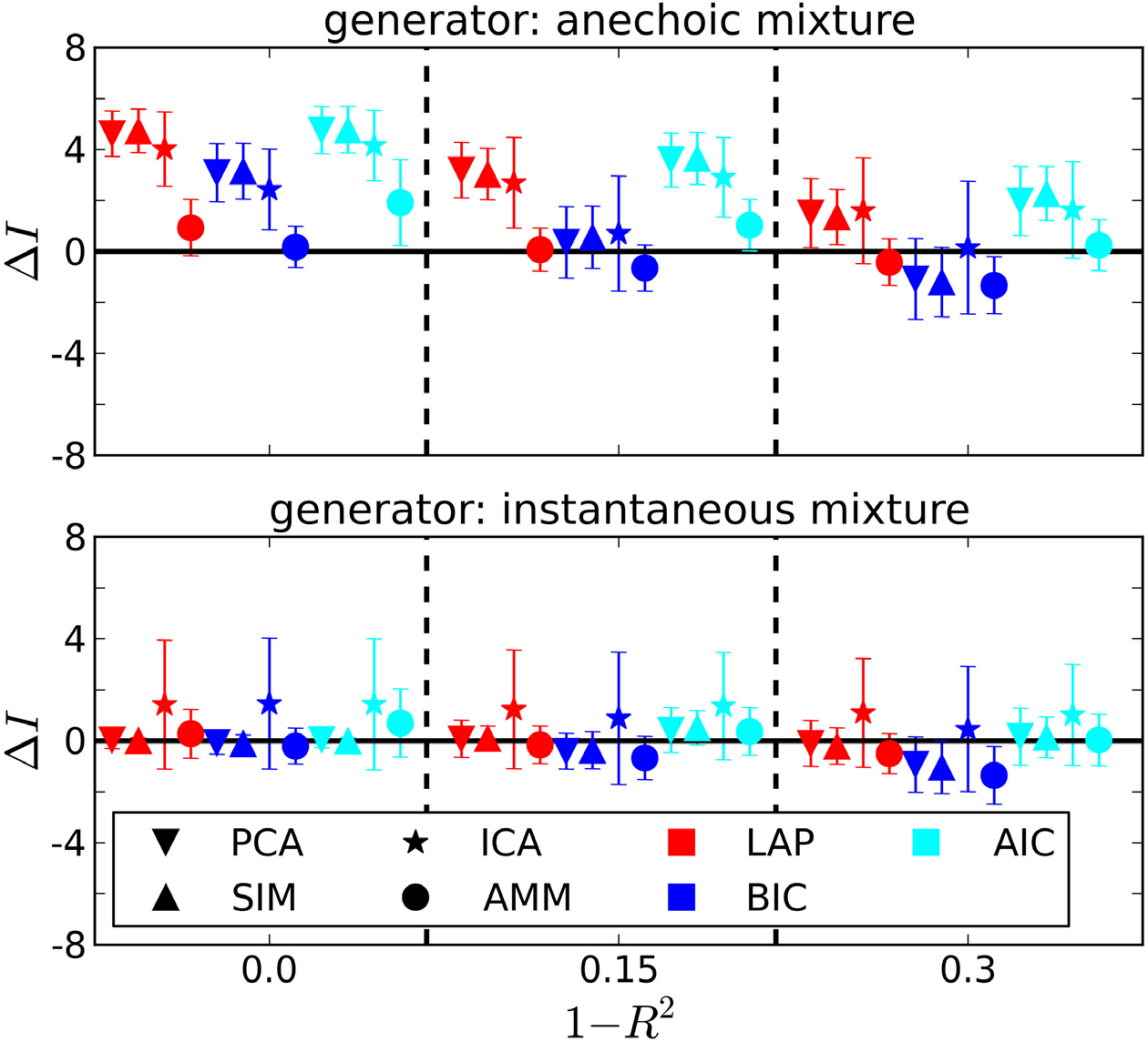
**Estimating the number of sources in the ground truth from *J* = 10 trials**. Δ*I* =estimated-generating number of sources. 1 − *R*^2^: noise level from Equation 41. Error bars are ± one standard deviation. Symbol shapes stand for analysis algorithms, colors indicate the selection criterion. **Top panel**: Anechoic generator. If an AMM is used for analysis, BIC and LAP perform comparably well within the error bars, AIC tends to overestimate. For incorrect analysis models (SIM/ICA/PCA), all criteria overestimate the number of sources, since the extra variability provided by the time shifts needs to be explained via additional sources in instantaneous mixture models. Note, however, that a model type determination step based on BIC or LAP would have ruled out an instantaneous mixture with high probability (cf. Figure 3 and Table 2). **Bottom panel**: For the instantaneous mixture (bottom panel) all three criteria give good results when using PCA or SIM models.

The dependency of the estimated number of sources on the noise level is depicted in Figure 4 for *J* = 10. Also unsurprisingly, the estimated number of sources decreases with increasing noise level, since noisy data contain less information about the generating process.

#### 3.1.3. Temporal smoothness constraints

**Figure 5:**
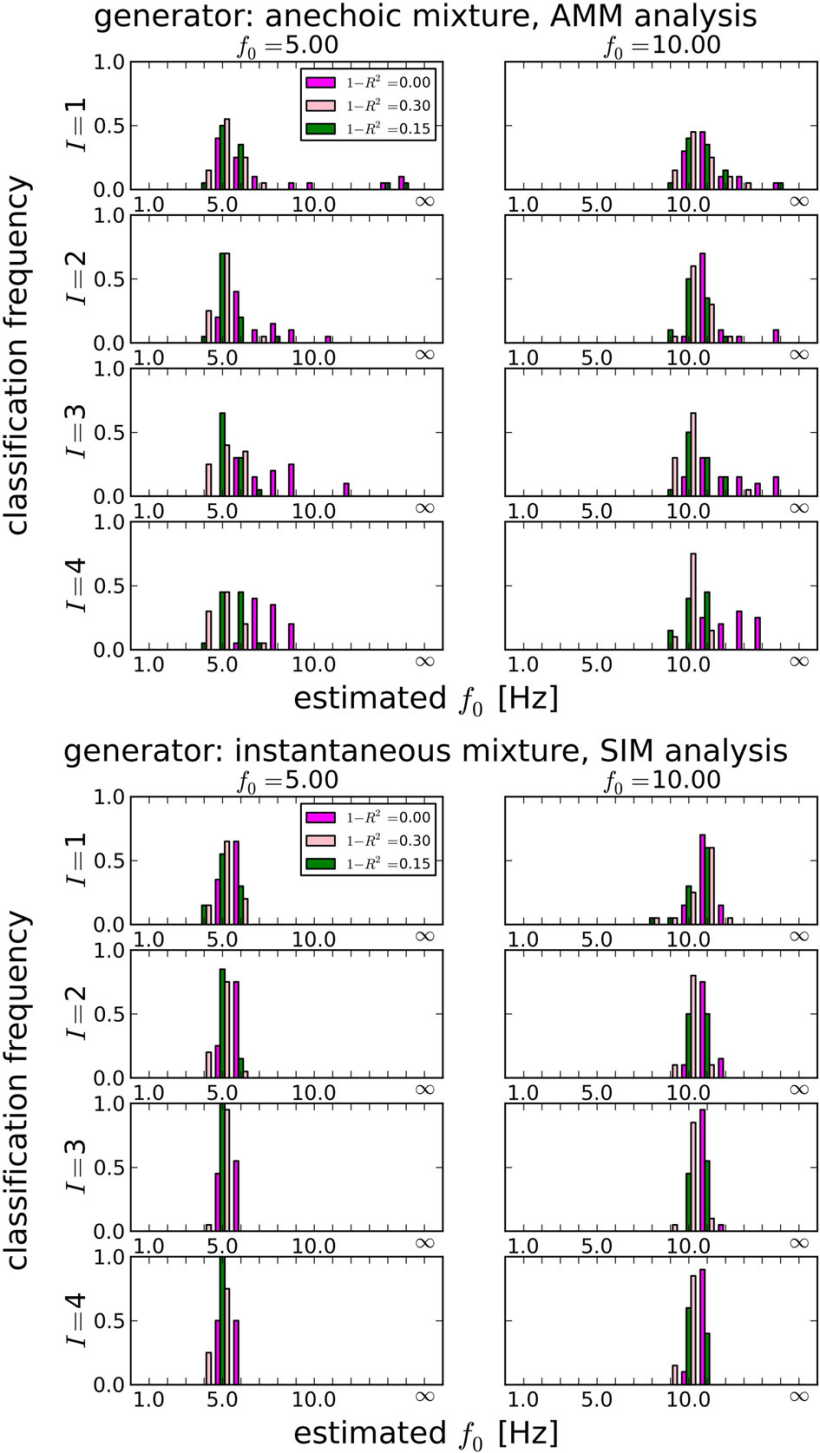
**Estimating the best temporal smoothness regularization cutoff frequency *f*_0_ (Equation 26) for *J* = 10 trials**. Results obtained with LAP criterion. This estimation can not be done with AIC or BIC: temporal smoothness, while it reduces the effective degrees of freedom (DF), cannot be expressed in BIC or AIC, because these criteria need an integer number for the DF, which would not change with a continuous regularization like smoothness. We tested 16 smoothness settings: {1Hz, 2Hz, …, 15Hz} and no smoothness constraint, indicated by “∞” in the plots (this is equivalent to a pPCA model). Left column: 5 Hz ground truth, right column: 10 Hz ground truth. Estimating the smoothness works well for both anechoic **(Top)** and instantaneous **(Bottom)** mixtures, except for the zero-noise anechoic case, where *f*_0_ is overestimated by ≈ 2 Hz on average.

In section 3.1.1, we showed that LAP is the only criterion which can detect the presence of temporal smoothness constraints. Now we investigate whether it can also identify the amount of smoothness, i.e., *f*_0_ in Equation 26. To this end, we computed the LAP score for 16 smoothness settings: {1Hz, 2Hz, …, 15Hz}, and also without smoothness constraint (i.e., effectively a pPCA model). We select the optimal smoothness setting for each dataset, and compute the average deviation to the generator smoothness (either 5 or 10 Hz) across all numbers of generating and estimated sources. The results are summarized in Table 3 for all noise levels, Figure 5 shows the detailed distributions for *J* = 10 trials. Except for the noiseless anechoic case, the correct temporal smoothness is found with average deviations near zero and standard deviations < 1.5Hz. We have as of yet no explanation for the overestimation in the noiseless anechoic case, but speculate that it is due to some jitter in the estimated delays of the anechoic model, which can be “explained away” by allowing for high-frequency components in the sources. As soon as noise is present in the data, this effect disappears.

**Table 3:** **Mean temporal smoothness estimation accuracies and standard deviations for LAP criterion, marginalized across number of source of both generator and analysis model**.

**1 − *R*^2^**			
**Gen**.	**0.00**	**0.15**	**0.30**
**NUMBER OF TRIALS *J*:5**			
SIM	0.526 ± 0.584	−0.171 ± 0.605	−0.526 ± 0.803
AMM	2.224 ± 1.793	0.137 ± 1.469	−0.553 ± 0.956
**NUMBER OF TRIALS *J*:10**			
SIM	0.694 ± 0.487	0.019 ± 0.518	−0.325 ± 0.638
AMM	1.762 ± 1.656	0.275 ± 1.475	−0.346 ± 1.441
**NUMBER OF TRIALS *J*:25**			
In	0.806 ± 0.494	0.275 ± 0.536	0.006 ± 0.553
An	2.150 ± 1.848	0.562 ± 1.288	0.106 ± 0.795

*Estimation accuracy is given by best estimated *f*_0_ (see Equation 26) as determined by LAP minus actual cutoff frequency (either 5 or 10 Hz). Gen. is the generative model: anechoic (AMM) or smooth instantaneous mixture (SIM). 1 − *R*^2^ is the noise level (Equation 41). Except for the zero-noise anechoic generator, *f*_0_ can be determined to within 1 Hz of its true value. For details, see text*.

### 3.2. Human gait analysis

**Figure 6:**
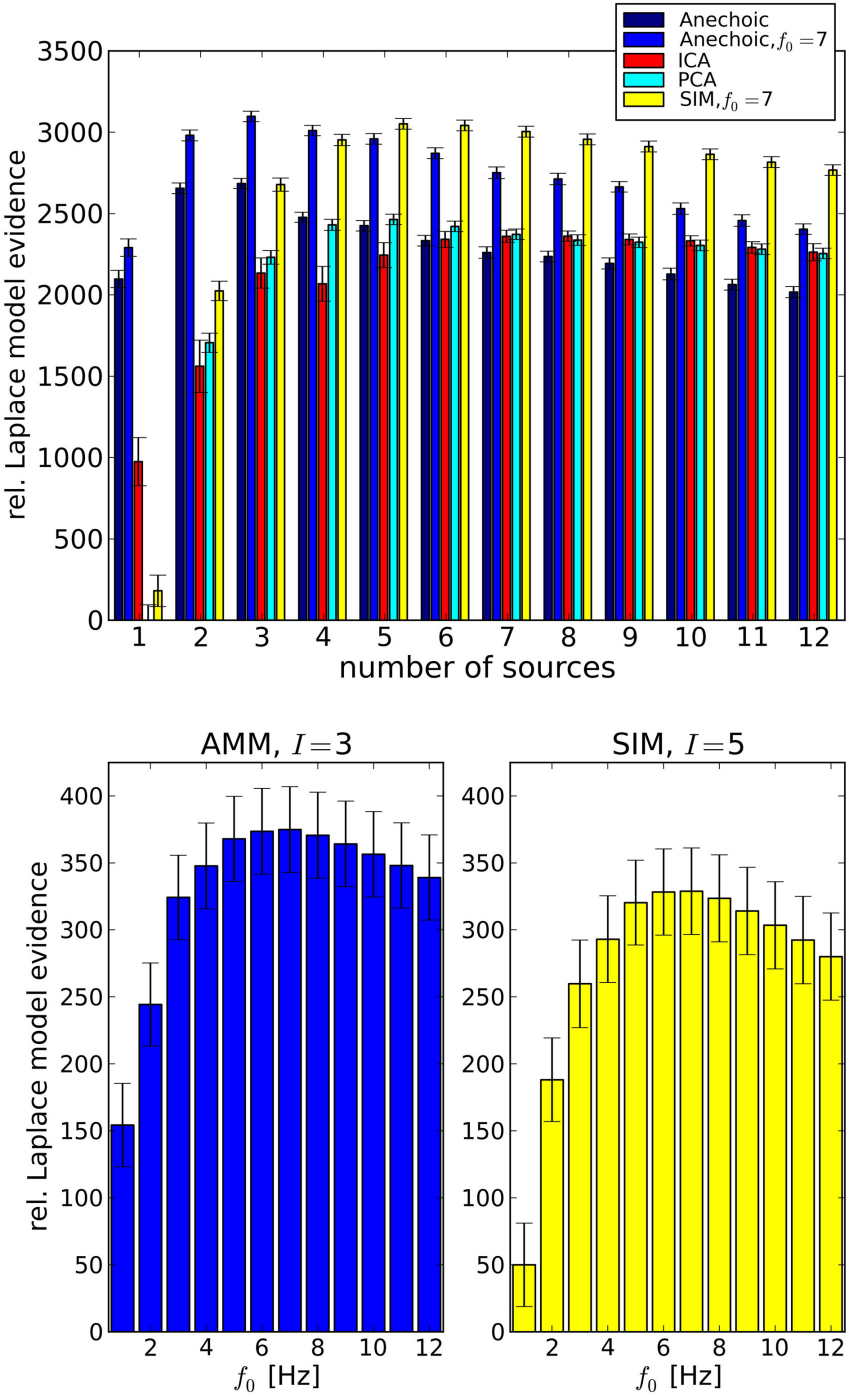
**Top**: Analysis of emotional gait data from Roether et al. (2008) with PCA, ICA and Anechoic demixing for different numbers of sources. The bars represent model evidences computed with Laplace approximation, relative to the lowest observed model evidence (PCA, 1 source). The anechoic analyses were carried out either without smoothing (black, *f*_0_ → ∞) or with the optimal *f*_0_ = 7 Hz (blue) for the wave kernel (see Equation 26). Error bars are standard errors, computed across trials. The best model (highest evidence) is the anechoic mixture with three sources and *f*_0_ = 7 Hz, followed by the SIM model with *f*_0_ = 7 Hz. PCA and ICA are significantly worse for any number of sources. **Bottom**: Detailed cutoff frequency analysis of the AMM model (left) and the SIM model (right), at their respective best number of sources *I*. The LAP score (relative to the SIM model with *I* = 1 set to 50) for both models peaks at *f*_0_ = 7Hz. However, the best AMM model's approximate posterior probability is larger than the best SIM posterior by a factor of ≈ 10^19^. For details, see text.

Having confirmed the validity of the LAP criterion on the synthetic ground truth, we now turn to real data. Since we are interested in smoothness properties as well as model types, we carried out a comparison between PCA and ICA; and SIM and AMM models with different *f*_0_ ∈ {1Hz; …; 12Hz}. The results are summarized in Figure 6, top, where the simple “Anechoic” model (dark blue) is an AMM without smoothness constraints. As might be expected, AMMs are the best models (within our tested models) for this kind of data. Furthermore, a correctly chosen *f*_0_ increases the LAP score significantly, i.e., the soft constraint provided by the smoothing kernel is an important feature of these kinematic data, see Figure 6, bottom. The best AMM has 3 sources, whereas the best SIM model needs 5, and has a lower score (see Figure 6, bottom).

LAP is an approximation of the marginal log-probability of the data [cf. Equation 3]. The best SIM model and the best AMM differ by a LAP score of ≈ 46, which translates into a probability ratio of $\frac{P\left(\text{AMM}\right)}{P\left(\text{SIM}\right)}>10^{19}$. The best PCA model (5 sources) has a LAP score which is lower than the 7 Hz AMM score by ≈ 600.

Note that individual datasets consisted of *J* = 8 trials (one per joint angle), therefore models with more than 8 sources are a priori too complex. This fact is also detected correctly by LAP, which assigns a roughly linearly decreasing score (exponentially decreasing in marginal probability) to models with ≥ 8 sources.

## 4. Discussion

In this study, we attempted to develop a more objective probabilistic criterion for motor primitive model selection. Our criterion turned out to be more reliable than other already existing classical criteria (cf. sections 1.1 and 3) in selecting the generative model underlying a given data set, as well as in determining the corresponding dimensionality. The criterion can moreover provide accurate information about soft constraints, here the smoothness of the temporal evolution of the signals.

We tested LAP performance on synthesized, kinematic-like data and on actual motion capture trajectories. However, motor primitives have also been identified at the muscle level (Bizzi et al., 2008), where usually the signals are rectified after collection. As we tested LAP only on data with unconstrained signs, its applicability to positive-only data, such as EMG recordings, is a subject for further investigations.

The application of the criterion to emotional gait trajectories suggested the anechoic model as the most suitable description of the data. This result is in agreement with previous findings from our lab (Omlor and Giese, 2007b), where it was demonstrated that the anechoic model can represent emotional gait data more efficiently (in terms of data compression) than other classical synchronous models. Also the best number of primitives determined by LAP is in line with Omlor and Giese (2007b), where three components were found capable to explain about 97% of the total data variation. Interestingly, the criterion suggested a temporal smoothness regularization with *f*_0_ = 7 Hz. Such a value may at first seem to be in contradiction with the step frequency of normal walking behavior that tends to be around 2 Hz (Pachi and Ji, 2005). The higher frequency value found by LAP can however be justified by multiple reasons. First, our data comprised also happy walks, which are known to be characterized by higher movement energy (Omlor and Giese, 2007b; Roether et al., 2009) and higher average movement velocity when compared to neutral or sad walks (Omlor and Giese, 2007a; Roether et al., 2009). Therefore, the average frequency power spectrum of the walking trajectories shows indeed considerable power within the band ranging from 0 to 10 Hz, with a peak at 5 Hz. In addition, in Figure 6 the maximum LAP score occurs at *f*_0_ = 7 Hz. However, taking the error bars into account, the LAP score associated with the optimal frequency is not statistically different from that associated with any score in the range [3 Hz,10 Hz], in agreement with the power spectrum. Another factor contributing to *f*_0_ = 7 Hz might be the tendency of LAP to overestimate the cutoff frequency slightly for nearly noise-free datasets, see Figure 5.

Additional and more advanced models of motor primitives, corresponding to a multivariate version of the anechoic mixture model considered in this study, have been developed (d'Avella et al., 2003, 2006) to describe the modular organization associated with EMG data sets. As we have not computed the LAP for these models, they are not among the possible model selection options yet. Future work will therefore aim to formulate the priors and generative models which would allow for the application of LAP to EMG data.

An interesting feature of LAP is its capability to discriminate between instantaneous vs. anechoic mixtures. The importance of introducing temporal delays in the model of a motor behavior has revealed to be crucial in some cases such as, for instance, in the modeling of emotional movements or facial expressions (Roether et al., 2008; Giese et al., 2012). LAP is to our knowledge the first model selection criterion explicitly designed for this.

Another remarkable feature of LAP is its capability, thanks to the addition a smoothness prior, to identify the amount of smoothness in the data, in other words to select the frequency *f*_0_ in Equation 26 based on the available data. While a Fourier analysis would also reveal where the power spectrum drops off, LAP has the advantage of providing a principled, quantitative trade-off between smoothness and goodness-of-fit, which allows for a more objective selection of *f*_0_. However, computing the power spectrum could be a first step to determine the range of *f*_0_s across which to search for the optimum.

Moreover, incorporating smoothness priors in time and/or space might be a viable extension of LAP to make it suitable to distinguish between a low-dimensional generative model based on time-invariant primitives vs. a model based on space-invariant primitives. Muscle synergies, for instance, have indeed been presented in the literature in those terms. Among them, “synchronous” synergies (Cheung et al., 2005; Ting and Macpherson, 2005; Torres-Oviedo et al., 2006) have been described as stereotyped co-varying groups of muscles activations, with the EMG output specified by a temporal profile determining the timing of each synergy during task accomplishment. This definition of synergies reflects the idea of invariance across space (namely the space spanned by the muscles) mentioned above. “Temporal” synergies (Ivanenko et al., 2004, 2005; Chiovetto et al., 2010, 2012), are instead defined as temporal activation profiles that can be simply linearly combined together to reconstruct the actual activity of each muscle. Such a definition of synergies is therefore incorporating a notion of invariance across time. Also more “hybrid” definition of primitives have been given. “Time-varying” synergies (d'Avella et al., 2003, 2006), for instance, are defined as spatio-temporal pattern of muscle activations, with corresponding EMG output determined by the scaling coefficients and time delays associated with each synergy. Chiovetto et al. (2013) already showed heuristically what movement features these definitions of synergies are describing. Although this knowledge can surely help to decide, dependent on the kind of analysis that one needs to carry out, which kind of synergies to extract from a given EMG data set, it however, does not provide a systematic criterion for such a decision. An extension of LAP might help here, too.

To apply LAP to a given source extraction method, it is necessary to (re)formulate this method in the language of generative probabilistic models. Only when the joint probability of the data and all latent variables (such as **W** or **S**) is available can Equation 38 be evaluated. Furthermore, since LAP results from a second-order approximation to the exponent of that joint probability, LAP will only yield (approximately) correct answers if such an approximation is valid. While the possibility of reformulating a given method can usually be decided *a-priori*, the validity of the second-order approximation typically needs testing on ground-truth data.

In conclusion, we presented an innovative and objective criterion that can be used to reliably select an adequate factorization model to explain the variance associated with kinematic/dynamic data and its corresponding dimensionality. We showed LAP to perform better than two plug-in estimators, BIC and AIC. It needs, however, to be extended to be used in the future for additional types of data, such as EMG data.

### Conflict of interest statement

The authors declare that the research was conducted in the absence of any commercial or financial relationships that could be construed as a potential conflict of interest.

## Footnotes

^1^We will refer to this model interchangeably by pPCA or just PCA in the following.
